# Effects of Three Types of Movements of Nickel–Titanium Instruments on Root Canal Preparation: Analysis by Using Cone-Beam Computed Tomography

**DOI:** 10.3390/ma18235417

**Published:** 2025-12-01

**Authors:** Kinga Kaczor-Wiankowska, Maciej Czechowski, Philipp Arndt, Aleksandra Joanna Wiankowska, Weronika Kwiecień, Katarzyna Lewusz-Butkiewicz

**Affiliations:** 1 Department of Conservative Dentistry and Endodontics, Pomeranian Medical University in Szczecin, 72 Powstancow Wielkopolskich St., 70-111 Szczecin, Poland; katarzyna.lewusz.butkiewicz@pum.edu.pl; 2 Student Scientific Circle at the Department of Conservative Dentistry and Endodontics, Pomeranian Medical University in Szczecin, 72 Powstancow Wielkopolskich St., 70-111 Szczecin, Poland; czechowskimaciej294@gmail.com (M.C.);

**Keywords:** cone-beam computed tomography, endodontics, root canal preparation, rotation

## Abstract

The development of endodontics leads to increasingly innovative techniques, which improve mechanical root canal preparation. Endostar E3 Azure (Poldent Co., Warsaw, Poland) is a nickel–titanium file, which can be used in rotary, reciprocal, and optimum torque reverse (OTR) movements. The aim of this study was to assess canal transportation (CT), canal-centering ability (CCA), and wall thickness reduction (WTR) after the use of Endostar E3 Azure files in these three movements. In total, 24 two-canal artificial teeth were used, which were divided into three groups, depending on the applied movement (n = 16 canals). Each canal was initially prepared manually and then instrumented with Endostar E3 Azure files using rotary, reciprocal, or OTR movements. Cone-beam computed tomography was performed before and after canal preparation. The root wall thickness was measured at 3 mm, 6 mm, and 9 mm from the radiological apex and CT, CCA, and WTR were calculated. Reciprocal movement resulted in significantly better outcomes in canal-centering ability (CCA = 0.57) compared with rotary movement (CCA = 0.27) in the middle part of the canal. The wall thickness was significantly reduced in the rotary group: 0.21, 0.19, and 0.13; in the reciprocal group: 0.09, 0.08, and 0.1; and in the OTR group: 0.11, 0.15, and 0.17 at 3, 6, and 9 mm from the apex, respectively. Moreover, rotary movement caused a statistically greater reduction in wall thickness in the apical and middle area compared to other groups. Endostar E3 Azure files significantly reduce the thickness of the root wall along its entire length, which may indicate the effective removal of infected tissue. The use of OTR movement did not affect the analyzed parameters negatively, and it is a safe option which combines the advantageous features of rotary and reciprocal movements.

## 1. Introduction

Mechanical preparation of the canal includes removing infected tissue, obtaining access for disinfecting fluids to penetrate the apical section of the root canal, creating sufficient space for obturation of the canal, whilst retaining the integrity of the root structure and maintaining the original shape of the canals [1,2]. It is optimal when the canal is sufficiently enlarged, and at the same time, significant preparation errors such as canal transportation (CT), which can lead to the formation of a ledge or perforation and weakening of the root tissue, are avoided [2,3]. The lower the canal transportation is, the smaller the deviation of the endodontic file in relation to the original axis of the canal, which is called canal-centering ability (CCA) [4]. The widening of the canal should decrease the closer the distance towards the apex, and safe wall thickness is approximately 0.3 mm [5] or 1 mm [6] after preparation. The thinner the thickness of the dentin, the higher the probability of complications occurring, e.g., vertical root fracture [7].

Endodontic treatment has experienced rapid growth and development, and it is associated with the usage of mechanical endodontic nickel–titanium files, which can operate with rotary, reciprocal, or optimum torque reverse (OTR) movements. The first nickel–titanium system engine-driven files moved in rotary movement, also known as continuous rotation, which consists of a clockwise up-and-down movement [2,8]. Reciprocal movement, which may be determined as repetitive up-and-down or back-and-forth movement, is a form of oscillating movement in which an instrument rotates in one direction, but before completing a full rotation cycle, the movement direction is reversed [2,9]. Compared with rotary movement, Aranguren et al. [10] reported that reciprocal movement extends the lifespan of an instrument by increasing its resistance to fatigue. Moreover, this movement allows one to avoid the pulled down movement into the canal effect [11] and has similar or better cutting efficacy than rotary movement [12]. On the other hand, reciprocal movement leads to greater debris accumulation than rotary movement [12]. OTR movement, also known as complex or torque-sensitive reciprocal rotation, involves rotary movement that transitions into reciprocal movement after exceeding the torque set in the endodontic motor: alternating 90° and 180° counterclockwise and clockwise rotation, respectively, upon exceeding a predetermined torque value [8,13,14]. The advantages of OTR movement are maintaining the cutting efficiency despite reciprocal movement, improving cyclic fatigue resistance and the extended lifespan of files [14,15,16,17]. However, the OTR movement does not reduce the risk of developing dentinal cracks [18]. Regardless of the movement used, the files should cause an optimal reduction in the root wall thickness (WTR), the least possible CT with the best possible CCA [19].

One of the newer endodontic nickel–titanium file systems is Endostar E3 Azure (Poldent Co., Warsaw, Poland), which is composed of martensite and austenite material. Martensite grants ensure high flexibility, adjustment to the curvature of the canal, and high resistance to instrument separation, resulting in a longer file lifespan, whereas the austenite part of the file provides elasticity, efficient cutting ability, and increased twisting resistance. This system is unique because it has been designed to work and operate with three types of movement: rotary, reciprocal, and OTR [8,13]. Endostar E3 Azure has a modified S-shape section with two 90-degree cutting edges. The file has variable pitches and variable helical angles, which decrease the risk of being pulled down into the canal, and increase better cutting efficiency and debris evacuation. Moreover, the inactive tip is safer for canal preparation, and minimizes the risk of canal transportation and perforation [8].

Despite the described advantages of the Endostar E3 Azure, studies that analyze the impact of this file system on canal preparation are limited. Therefore, the authors decided to conduct research using this system. This is the first study to analyze selected parameters of root canal preparation, using one type of file in three different types of movements. The aim of this study was the assessment of CT, CCA, and WTR after using Endostar E3 Azure in rotary, reciprocal, and OTR movements. The first null hypothesis states that there was no significant difference in CT, CCA, and WTR while using Endostar E3 Azure in rotary, reciprocal, and OTR movements. The second null hypothesis recognizes no difference in the preparation of the root canal along its entire length.

## 2. Materials and Methods

### 2.1. Sample Size Calculation

Sample size calculation was performed on the dentin thickness reduction data from Gagliardi et al. [20], where a power of 0.95 with an effect size of 0.804 and a significance level of 0.05 was achieved, showing that an adequate sample size was used in this study.

### 2.2. Specimen Preparation

In this study, 24 two-canal artificial upper premolars were used, which were divided into 3 groups depending on the movement of the file, with 8 teeth per group (n = 16 canals). The artificial teeth were selected based on the following inclusion criteria: the canals were patent-tested with an ISO 10.02 instrument (Poldent Co., Warsaw, Poland), its length was 21.0 mm ± 0.5 mm, and they were straight (≤5°). The working length for each canal was established from the apical reference point, which was 0.5 mm from the apex, to the coronal reference point, which was at the top of the cusps. The glide path was prepared manually using K-files 10.02. 15.02, and 20.02. Next, all canals were shaped using the Endostar E3 Azure Basic (Poldent Co., Warsaw, Poland) set of instruments—30.08 (used in the ½ coronal part of the canal), 25.06, and 30.04—which were used throughout the whole working length, with different movements: control group—rotary (it was the first to be introduced into the manufacturing of engine-driven nickel–titanium files)—and experimental group—reciprocal or OTR. Each pair of artificial canals was prepared with a new set of individual instruments, which included a K-file (Poldent Co., Warsaw, Poland) ISO 10.02, 15.02, and 20.02 and a set of Endostar Azure E3 Basic. During instrumentation, each canal was irrigated with 5.25% NaOCl. Instrumentation with rotary and reciprocal movements was performed with an X-Smart Plus endodontic micromotor (Dentsply Sirona, York, PA, USA), whereas instrumentation with OTR movement was performed with an Endostar Provider (Poldent, Warsaw, Poland). The torque and speed of the file movements were set according to the manufacturer’s instructions [8,13]:

Control group—rotary movement: the instruments rotated clockwise with a set speed of 300 rpm and a 2.4 Ncm torque, with up-and-down movement.

Experimental group—reciprocal movement: the instruments performed alternating clockwise and counterclockwise movements—150 degrees clockwise and 30 degrees counterclockwise movements.

Experimental group—OTR movement: It combined the rotary and reciprocal movements. The files rotated with a set speed of 300 rpm and 1.0 Ncm torque until excessive torque resistance was reached. When the file exceeded the torque, it started to rotate 90 degrees to the left and then 180 degrees to the right and continued reciprocal movement until resistance dropped, as recommended by the manufacturer. Sample preparation and storage were performed under room temperature and humidity.

### 2.3. Cone-Beam Computed Tomography (CBCT) Scanning and Slice Analysis

**Figure 1:**
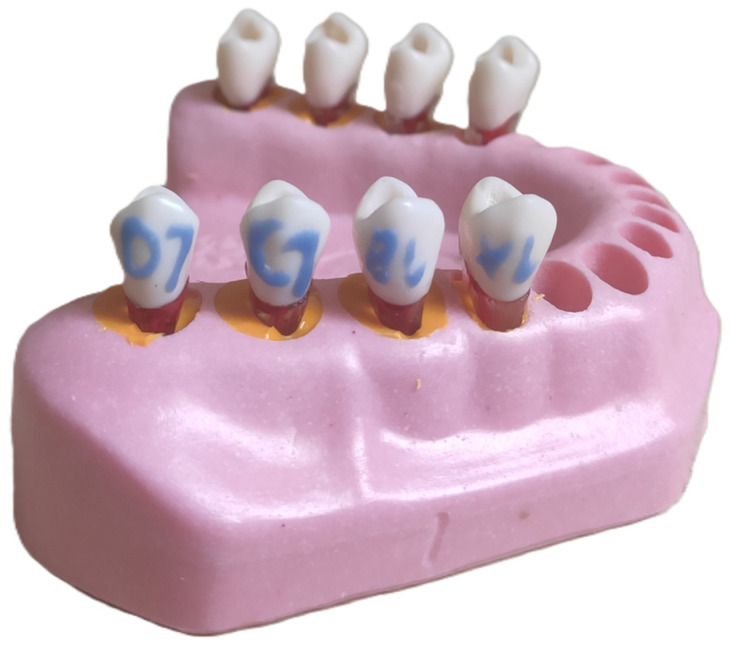
Samples put into a plastic model.

The samples were placed in a plastic model and fixed with A-polyvinyl siloxane material in order to perform CBCT imaging before and after canal preparation (Aquasil Ultra XLV, Dentsply Sirona, York, PA, USA) (Figure 1). To achieve a repeatable position of the samples, they were placed in the plastic model with their own position, and a different plastic model was assigned to each group. Furthermore, samples were stored at room temperature and the samples were removed from the models only to prepare the canals.

**Figure 2:**
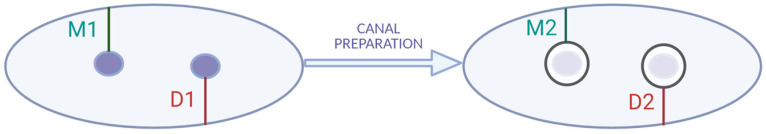
Schematic representation of measurements of root thickness before (M1, D1) and after (M2, D2) canal preparation.

CBCT scanning was performed using a NewTom GiANO HR scanner (Cefla s.c., Bologna, Italy) at 90 kV, 6 mA, 21.6 s of total scan time, with a voxel size of 0.3 mm and an 8 cm × 10 cm field of view. The obtained CBCT horizontal slices were analyzed via ImageJ 1.54 software (National Institutes of Health, Bethesda, MD, USA) to estimate the following values at 3 mm, 6 mm, and 9 mm from the radiological apex, which corresponds to the apical, middle, and coronal parts of the root:

M1—The shortest distance from the mesial external surface of the sample’s root to the mesial wall of the non-instrumented canal (Figure 2).

M2—The shortest distance from the mesial external surface of the sample’s root to the mesial wall of the instrumented canal (Figure 2).

D1—The shortest distance from the distal external surface of the sample’s root to the distal wall of the non-instrumented canal (Figure 2).

D2—The shortest distance from the distal external surface of the sample’s root to the distal wall of the instrumented canal (Figure 2).

The values of M1, M2, D1, and D2 were estimated by a single operator (K.K-W), and then used to calculate the CT and CCA according to the formula of Gambill et al. [19]. CT was calculated using the following formula: CT = (M1 − M2) − (D1 − D2). When CT equals zero, no canal transportation is present, a value below zero means transportation in the distal direction, and a value greater than zero means transportation in the mesial direction [19]. The canal-centering ability ratio was calculated using the following formula: CCA = (M1 − M2)/(D1 − D2) or CCA = (D1 − D2)/(M1 − M2). A smaller value was considered if the results of these two formulas were different. A CCA value of one indicates perfect canal-centering ability. The closer the CCA value is to zero, the worse this parameter is [19]. The last parameter was WTR, which was calculated using the following formula: WTR = 1 − (M2 + D2/M1 + D1).

### 2.4. Statistical Analysis

The statistical analysis was performed using Statistica 13.3 software (StatSoft, Inc., Tulsa, OK, USA). The normality of the data was assessed using the Shapiro–Wilk test. The CT and WTR data were found to be normally distributed and were analyzed using ANOVA and post hoc tests. A paired *t* test was used for intragroup analyses of wall thickness in the same region of the canal, before and after preparation. The normality of the CCA data was not observed, and the Kruskal–Wallis and post hoc tests were used. The following post hoc tests were used for pair comparisons if the ANOVA (for WTR and CT analyses) or the Kruskal–Wallis (for CCA analysis) test was significant: Tukey’s test after ANOVA and Dunn’s test after Kruskal–Wallis. The level of significance was established as *p* = 0.05 for all tests.

## 3. Results

There was no file separation during sample preparation. The minimum, maximum, mean, and standard deviation of the CT and CCA values are shown in Table 1 and Table 2, respectively. Intragroup analysis showed that there were no statistically significant differences in the same group between apical, middle, and coronal parts of the canal, both in CT (Table 1) and CCA (Table 2).

Only OTR movement caused distal transportation of the canal in the apical (CT = −0.02) and coronal (CT = −0.015) parts of the canal, but this difference was not statistically significant. Comparing the movement of the files, there were no statistically significant differences in the CT values across the entire length of the canals (3 mm: *p* = 0.543; 6 mm: *p* = 0.385; 9 mm: *p* = 0.971). The best CCA in the coronal part of the canal was shown by files used in the control group (CCA = 0.47) and OTR (CCA = 0.51) movements, as opposed to reciprocal movement (CCA = 0.39), but these results were not statistically significant. Compared with the movements of the files, the CCA values were significant only in the middle part of the canal (3 mm: *p* = 0.305; 6 mm: *p* = 0.044; 9 mm: *p* = 0.644). A post hoc test showed that the CCA in the middle part of the canal differed significantly between reciprocal and control groups (*p* = 0.017).

The thickness of the root wall before and after canal preparation and the WTR results are shown in Table 3.

**Figure 3:**
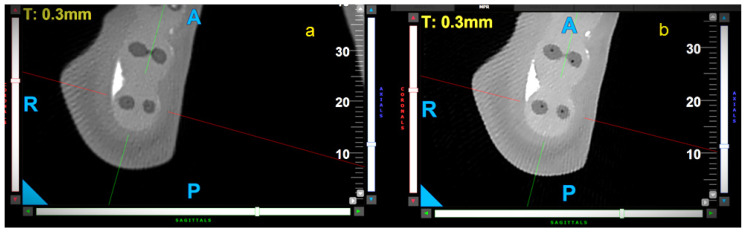
Representative CBCT axial—horizontal slices of samples at 3 mm from apex, before (**a**) and after (**b**) canal preparation—rotary movement. The letters A, P, R indicate sides—anterior, posterior, and right respectively.

**Figure 4:**
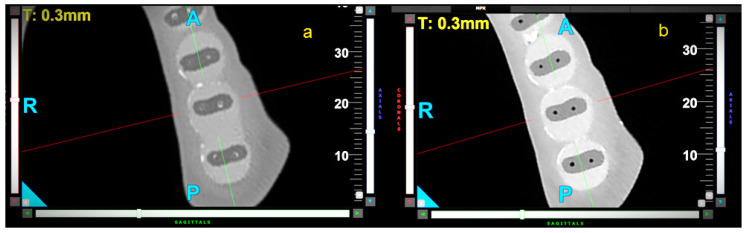
Representative CBCT axial—horizontal slices of samples at 6 mm from apex, before (**a**) and after (**b**) canal preparation—reciprocal movement. The letters A, P, R indicate sides—anterior, posterior, and right respectively.

**Figure 5:**
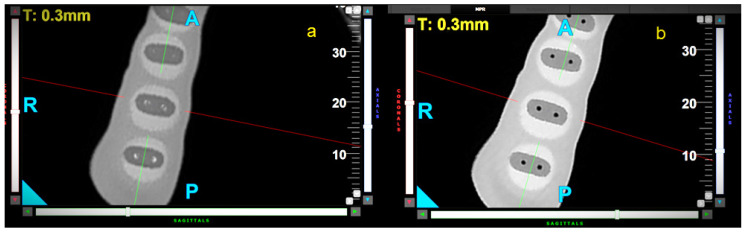
Representative CBCT axial—horizontal slices of samples at 9 mm from the apex, before (**a**) and after (**b**) canal preparation—OTR movement. The letters A, P, R indicate sides—anterior, posterior, and right respectively.

All file movements resulted in a significant reduction in wall thickness over the entire length of the samples. The control group presented the highest WTR in the apical (WTR = 0.21) and middle (WTR = 0.19) parts of the samples. The differences between the movements were statistically significant in the apical and middle parts of the root (3 mm: *p* < 0.001; 6 mm: *p* = 0.007). OTR movement caused the highest WTR in the coronal part of the samples (WTR = 0.13), but the differences between these movements were not statistically significant (*p* = 0.056). Representative CBCT horizontal slices of samples at 3, 6, and 9 mm from the radiological apex are shown in Figure 3, Figure 4 and Figure 5.

## 4. Discussion

This is the first study that analyzes CT, CCA, and WTR after using Endostar E3 Azure files in three different movements using CBCT. Rebeiz et al. [21] reported that CT after the use of Endostar E3 Azure files with reciprocal movement at 5 mm and 7 mm from the apex was significantly less than rotary movement; however, their study did not analyze OTR movement.

In this study, artificial upper premolars were used. Previous studies have successfully used artificial samples in similar analyses [21,22,23,24]. Predictable anatomy, easy market access, low price, and good imitation of natural tissue are factors increasing their popularity in experimental studies [25]. Moreover, artificial canals have standardized dimensions, such as the angle and radius of the canal curvature, diameter, and length, which provide reliable data [22,26]. On the other hand, even the best artificial material will never be as adequate as natural organic structures. Nassri et al. [25] emphasize that the artificial material, which simulates pulp, has a higher viscosity compared to natural pulp, making it more difficult to remove than in reality. On the other hand, the hardness of the resin of artificial teeth is lower than natural tissues, and it may give the misleading impression of less resistance during root canal preparation than actually exists in clinical practice. Artificial teeth demonstrate lower-level contrast under radiological examination than natural tissue; however, the differentiation of the soft tissue-simulating material from the hard dental tissue-simulating material can be easily achieved [25]. However, the anatomy of natural teeth is not as predictable and poses a significant limitation in standardized experimental research. Moreover, biomaterial is harder to obtain, raising ethical questions, especially when tooth extraction becomes less popular [25]. In this research, upper premolars were used because this group of teeth and the mesial root of lower molars are most susceptible to vertical root fracture [27]. According to previous studies, increased dentine removal during endodontic treatment could lead to decreased fracture resistance of the tooth [28]. Additionally, the minimal recommended residual dentine thickness after root endodontic treatment or post preparation is 1 mm around the entire circumference of the canal [6]. Mesial and distal premolar wall thickness modifications were analyzed because the mesiodistal diameter of premolar roots is narrower than the buccolingual diameter. Overpreparation of these walls may increase the risk of canal perforation or weaken root canal walls [29]. In this study, rotary movement was classified as a control group because it was the first to be introduced into the manufacturing of engine-driven nickel–titanium files [2]. Moreover, it is the most widespread and more frequently introduced by manufacturers of new nickel–titanium files compared with the other two movements, reciprocal and OTR, which were classified as experimental groups.

The analysis of this study was performed using CBCT. The authors decided to use 8 cm × 10 cm field of view, because they aimed to obtain scans of all samples from one group in one CBCT. In spite of the authors performing CBCT with a voxel size of 0.3 mm instead of a smaller size, e.g., 0.2 or 0.15 mm, previous studies demonstrated that a bigger voxel size does not influence linear measurement detail [30,31]. It should be noted that increasing the voxel size could reduce image sharpness, but the final measurement quality is also influenced by, e.g., measurement software and CBCT devices. On the other hand, in a clinical aspect, increasing the voxel size leads to a reduction in radiation dose [31,32]. In order to improve the quality of measurements, an additional ImageJ software was used in this study. The lack of clear guidelines regarding the parameters of CBCT such as field of view, voxel size, and exposure parameters for laboratory tests could disrupt and complicate the comparisons of findings from different studies [33]. Therefore, it would be advisable to standardize imaging in laboratory studies. The analyses were performed using CBCT, which enables both quantitative and qualitative evaluation, without destroying the samples. Furthermore, the data obtained from CBCT are more advanced compared to traditional methods, such as periapical radiography. Moreover, three-dimensional images allow for the analysis of samples from various angles, which is crucial for a comprehensive assessment of structures, and is an accurate method for wall thickness, CT, and CCA measurements [34,35]. However, in some cases, high-density materials such as root canal sealers and interradicular posts may lead to lower image quality and contrast. This limitation might make measuring the remaining dentine thickness difficult [29]. Alternatively, specimen analysis could be performed using micro-computed tomography, as it allows a more detailed two- or three-dimensional analysis of the root canal anatomy, as well as its preparation and tissue loss [3,36,37,38].

The first null hypothesis was partially rejected because of significant differences in the middle and apical parts of the canal. The gathered results of this study show a statistical difference in the CCA between the control group and the group with reciprocal movement at 6 mm (Table 2). Agarval et al. [39] obtained similar results in the apical part of the canal and different results in the middle and coronal parts of the canal. Their study revealed no statistically significant difference in the CCA parameter in the middle part of the canal, and a significantly lower CCA parameter in ProTaper files (Dentsply Sirona, York, PA, USA)—rotary movement compared to WaveOne files (Dentsply Sirona, York, PA, USA)—reciprocal movement in the coronal part of the canal [39]. This could be explained by different file system properties, i.e., taper and flexibility differences between the E3 Azure files, WaveOne files, and ProTaper files, as well as different NiTi alloy preparations. Kyaw et al. [15] achieved similar results, when they evaluated the canal-centering ability and additional canal volume changes after using nickel–titanium files in rotary and OTR movements. Their study revealed no statistically significant differences between these two movements in 300 and 500 rpm speed [15]. Partially different results were obtained by Omori et al. [40], who compared ProTaper Universal (Dentsply Sirona, York, PA, USA) and ProTaper Gold (Dentsply Sirona, York, PA, USA) files in the three analyzed movements. They stated that reciprocal and OTR movements achieved significantly better CCA results compared to rotary movement at 0 mm from the apex for ProTaper Universal files and no significant differences between analyzed movements at 3 mm from the apex for both endodontic files.

Analyzing the CT parameter, no statistically significant differences were found between movements and regions in the same groups. The CT results in the apical area are identical to those of Rebeiz et al. [21], where the Endostar E3 Azure system in rotary and reciprocal movements were analyzed. Moreover, Romeo et al. [38] obtained approximately the same CCA and CT results as in our studies. They analyzed rotary, reciprocal, OTR, and additionally the Jeni movements digital assistance system, integrated with Canal Pro Jeni motor (Coltene/Whaledent AG, Alstätten, Switzerland), and did not notice statistically significant differences in CCA and CT between these movements in the apical, middle, and coronal parts of the canal [38].

Compared with other movements, rotary movement caused a significantly greater WTR in the middle and apical parts (Table 3). Similar results were achieved by Giuliani V et al. [41], who compared the ProTaper Universal file system in rotary and reciprocal movements and the WaveOne file system in reciprocal movement. They concluded that the ProTaper Universal system used with rotary movement removed a significantly greater amount of resin from artificial canals compared to reciprocal systems [41]. The opposite results were reported by Chaudhary et al. [42], who analyzed the remaining dentin thickness after preparation using the Mtwo (VDW, Munich, Germany) rotary movement and WaveOne file systems. Their study suggested that rotary files provide sufficient preparation without causing dangerous lowering of the dentine wall thickness. In contrast to our results, reciprocal movement caused greater dentin loss than rotary movement, but the results were not statistically significant. Other conclusions from Chaudhary et al. [42] may result from the different endodontic file system regarding their properties and different samples (natural anterior teeth versus artificial premolars) in their study.

The second null hypothesis was accepted because all of the movements caused a significant reduction in wall thickness throughout the entire length of the canal (Table 3). Proper root canal preparation allows better flow of disinfecting fluids and proper obturation; however, excessive wall preparation might increase the risk of weakening the structures [1,2]. In this study, none of the walls had a thickness of less than 1 mm after canal preparation. Similar results were obtained by Cerqueira et al. [43], who analyzed the changes in canal volume and wall thickness after canal preparation using XP-endo Shaper (FKG Dentaire SA, La Chaux-de-Fonds, Switzerland) and ProTaper Next (Dentsply Sirona, York, PA, USA) files. They reported that both files provided a significant reduction in wall thickness and an increase in canal volume without exceeding safe values. Moreover, Mangal et al. [44] analyzed dentin thickness after canal preparation using Mtwo files and reported that all analyzed walls in the middle and coronal parts of the canal were reduced but did not reach the critical thickness of 1 mm. Despite the significant reduction in wall thickness in all groups, there were no significant differences between regions in intragroup analysis (Table 3). Regardless of the movement used, the Endostar E3 Azure system prepares the canal in a uniform way along its entire length. Similar conclusions were achieved by Jaggi et al. [45], who analyzed rotary TruNatomy files (Dentsply Sirona, York, PA, USA) and reciprocal WaveOne file systems. In this study, there was no significant difference between the remaining dentin thickness in the coronal, middle, and apical parts of the canal according to the intergroup analysis.

Among the limitations of this study was the use of artificial samples. For this reason, it is advisable to extend these studies to in vitro studies with extracted teeth or in vivo studies. Although artificial teeth provide accurate and repeatable anatomy for research purposes, they do not mimic all the properties of natural, biological structures such as dentin. The repeatability of the anatomy can also be perceived as a significant limitation to the experiment, as notable anatomical differences may appear across the general population. This experimental research was also restricted to one file system. A comparison between two or multiple systems would be ideal, hence leaving space for future investigations and elaborations.

The lack of statistically significant differences between the three movements in the analysis of WTR and CT allows dentists to omit this aspect when choosing an endodontic file system. Clinicians should consider other properties of files, such as fracture resistance or their lifespan, when deciding which system should be used.

## 5. Conclusions

Regardless of the movement applied, Endostar E3 Azure files significantly reduce the thickness of the root wall along its entire length, which may enhance infected tissue removal during endodontic treatment. On the other hand, it should be remembered that excessive reduction in wall thickness can lead to root perforations or fractures. In addition, rotary movement significantly increases the amount of tissue removed from the apical and middle parts of the root canal, compared to reciprocal and OTR movements. The use of OTR movement did not affect the analyzed parameters negatively; therefore, it can be used successfully as a potentially safe and balanced option, which combines the advantageous features of reciprocal and rotary movements. Endostar E3 Azure files can be successfully used in everyday dental practice. They provide both safe and effective root canal preparation. Slightly better canal preparation parameters were achieved when using reciprocal or OTR movements compared to rotary movement, which may indicate the recommended movements for dental practice. The authors emphasize that these studies were conducted on artificial samples and should be confirmed in natural teeth as well as samples with other morphologies.

## Figures and Tables

**Table 1:** Canal transportation (CT).

	Group	Region (mm)	Min.	Max.	Mean ± SD	*p*
Control	Rotary	3	−0.43	0.57	0.07 ± 0.28	0.165
		6	−0.28	0.90	0.20 ± 0.29	
		9	−0.64	0.40	0.01 ± 0.28	
Experimental	Reciprocal	3	−0.74	0.76	0.03 ± 0.35	0.594
		6	−0.3	0.46	0.07 ± 0.18	
		9	−0.28	0.43	0.001 ± 0.25	
	OTR	3	−0.31	0.24	−0.02 ± 0.17	0.571
		6	−0.67	0.49	0.07 ± 0.39	
		9	−0.41	0.40	−0.015 ± 0.21	

**Table 2:** Canal-centering ability (CCA).

	Group	Region (mm)	Min.	Max.	Mean ± SD	*p*
Control	Rotary	3	0	0.9	0.31 ± 0.27	0.173
		6	0	1	0.27 ± 0.30 *	
		9	0	1	0.47 ± 0.34	
Experimental	Reciprocal	3	0.07	1	0.46 ± 0.28	0.336
		6	0.06	1	0.57 ± 0.36 *	
		9	0.01	1	0.39 ± 0.33	
	OTR	3	0.015	1	0.39 ± 0.32	0.55
		6	0.03	1	0.39 ± 0.35	
		9	0.1	0.94	0.51 ± 0.32	

* indicates statistically significant differences: *p* = 0.017; Cohen’s d = 0.906.

**Table 3:** Wall thickness and wall thickness reduction (WTR).

	Group	Region (mm)	Wall Thickness (mm)				WTR	*p*
			Before	After	Difference	*p*		
Control	Rotary	3	1.77 ± 0.23	1.38 ± 0.16	0.39 ± 0.22	<0.001	0.21 ± 0.11 ^A,B^	0.059
		6	2.11 ± 0.31	1.7 ± 0.24	0.41 ± 0.23	<0.001	0.19 ± 0.09 ^C^	
		9	2.4 ± 0.28	2.07 ± 0.19	0.33 ± 0.23	<0.001	0.13 ± 0.09	
Experimental	Reciprocal	3	1.43 ± 0.27	1.3 ± 0.23	0.13 ± 0.13	0.001	0.09 ± 0.08 ^A^	0.836
		6	1.73 ± 0.3	1.57 ± 0.2	0.16 ± 0.16	0.002	0.08 ± 0.08 ^C^	
		9	2.18 ± 0.3	1.95 ± 0.21	0.23 ± 0.17	<0.001	0.1 ± 0.07	
	OTR	3	1.35 ± 0.24	1.18 ± 0.18	0.16 ± 0.14	<0.001	0.11 ± 0.09 ^B^	0.202
		6	1.81 ± 0.28	1.51 ± 0.14	0.3 ± 0.25	<0.001	0.15 ± 0.09	
		9	2.16 ± 0.28	1.77 ± 0.22	0.39 ± 0.24	<0.001	0.17 ± 0.1	

Mean ± SD. The same uppercase superscript in WTR results indicates a significant difference between groups. ^A^: *p* < 0.001, Cohen’s d = 1.25; ^B^: *p* < 0.001, Cohen’s d = 0.99; ^C^: *p* = 0.007, Cohen’s d = 1.29.

## Data Availability

The original contributions presented in this study are included in the article. Further inquiries can be directed to the corresponding author.

## Abbreviations

The following abbreviations are used in this manuscript:

CT	Canal Transportation
CCA	Canal-Centering Ability
OTR	Optimum Torque Reverse movement
WTR	Wall Thickness Reduction
CBCT	Cone-Beam Computed Tomography
